# Bacterial Alkyl-4-quinolones: Discovery, Structural Diversity and Biological Properties

**DOI:** 10.3390/molecules25235689

**Published:** 2020-12-02

**Authors:** Muhammad Saalim, Jessica Villegas-Moreno, Benjamin R. Clark

**Affiliations:** School of Pharmaceutical Science and Technology, Tianjin University, 92 Weijin Road, Tianjin 300092, China; msaalim@tju.edu.cn (M.S.); q.jvillegas@gmail.com (J.V.-M.)

**Keywords:** quinolones, quorum sensing, *Pseudomonas aeruginosa*

## Abstract

The alkyl-4-quinolones (AQs) are a class of metabolites produced primarily by members of the *Pseudomonas* and *Burkholderia* genera, consisting of a 4-quinolone core substituted by a range of pendant groups, most commonly at the C-2 position. The history of this class of compounds dates back to the 1940s, when a range of alkylquinolones with notable antibiotic properties were first isolated from *Pseudomonas aeruginosa*. More recently, it was discovered that an alkylquinolone derivative, the Pseudomonas Quinolone Signal (PQS) plays a key role in bacterial communication and quorum sensing in *Pseudomonas aeruginosa*. Many of the best-studied examples contain simple hydrocarbon side-chains, but more recent studies have revealed a wide range of structurally diverse examples from multiple bacterial genera, including those with aromatic, isoprenoid, or sulfur-containing side-chains. In addition to their well-known antimicrobial properties, alkylquinolones have been reported with antimalarial, antifungal, antialgal, and antioxidant properties. Here we review the structural diversity and biological activity of these intriguing metabolites.

## 1. Introduction

The bacterial alkylquinolones are a class of microbial metabolites consisting of a 4-quinolone core, typically substituted with alkyl groups, most often at the 2 position [1]. The best-known of these are the Pseudomonas Quinolone Signal (PQS, **O7**), a potent modulator of quorum sensing behaviour in the *Pseudomonas* genus, and its biosynthetic precursor, 4-hydroxy-2-heptylquinoline (HHQ, **H7**). The discovery of these alkylquinolones begin with the use of a *Pseudomonas aeruginosa* (*Bacillus pyocyaneus*) extract by Bouchard for the prevention of anthrax in rabbits [2]. Emmerich and Low later demonstrated the antibiotic activity of the cell-free extracts of *P. aeruginosa* and named the antibiotic extract ‘pyocynase’, because at that time the antibiotic activity was attributed to the presence of enzymes [3]. Several years later Hays et al. isolated the actual antibiotic substances from *P. aeruginosa* and confirmed that they were small molecules. The names Pyo Ib, Ic, II, III and IV were proposed, and their antibiotic activity was demonstrated [4], though it took several years for the exact structures of these compounds to be determined. After studies involving chemical degradation, UV spectrophotometry, and total synthesis, the Pyo compounds were identified as alkylquinolones [5,6].

Pyo Ib and Ic were identified as 2-heptyl-4-quinolone (**H7**) and 2-nonyl-4-quinolone (**H9**) respectively [6], while Pyo-III was identified as 2-(Δ′1-nonenyl-)-4-quinolone (**H9Δ2**) [6]. Some of the Pyo compounds were in fact mixtures of several closely-related compounds: Pyo-II was determined to be a mixture of *N*-oxides: namely, 2-heptyl-4-quinolone-*N*-oxide (**N7**), 2-nonyl-4-quinolone-*N*-oxide (**N9**) and 2-undecyl-4-quinolone-*N*-oxide (**N11**) [6,7], while Pyo-IV was a mixture of the tetrahydroquinoline-2,4-diones **T7** and **T9** [8,9]. Budzikiewicz et al. isolated 2-*n*-(3-undecenyl)-4-quinolone (**H11Δ3**) from *P. aeruginosa* in 1979 and named it Pyo-V [10]. Over the course of the coming years, additional alkylquinolones were isolated from plant and microbial sources, often in a species-specific manner. Several of these alkylquinolones were named ‘pseudans’ because they were predominantly produced by *P. aeruginosa* [11].

Initially, only the antimicrobial activity of these molecules was the focus of attention, but in 1999 Pesci et al. made a breakthrough when they found that cell-cell communication in *P. aeruginosa* was not solely a function of the homoserine lactones. They isolated 3-hydroxy-2-heptylquinolone (**O7**) and named it the Pseudomonas Quinolone Signal (PQS), describing its activity as an auto-inducer, acting as a pivotal element in the quorum sensing system [12]. Later, other alkylquinolones responsible for quorum sensing were also identified [13]. It was also discovered that *P. aeruginosa* was not the only microbial species capable of producing alkylquinolones: bacteria belonging to *Alteromonas, Pseudoaltermonas* and *Burkholderia* genera have been shown to produce compounds from this class: so far, more than 57 alkylquinolones have been isolated from different sources. The Pseudomonas Quinolone Signal itself has been recently reviewed in detail [14], however, the properties of the wider alkylquinolone class are less well-described. Thus, in this review, we take a look at the microbial alkylquinolones in general, their structural diversity, bioactivity and their role in quorum sensing.

## 2. Structural Diversity and Distribution

**Figure 1:**
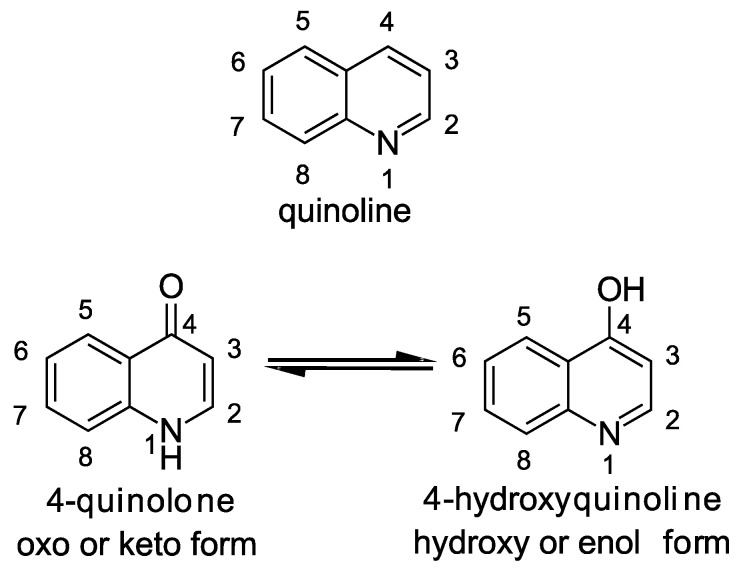
Chemical structures of quinoline and 4-quinolone scaffolds.

**Figure 2:**
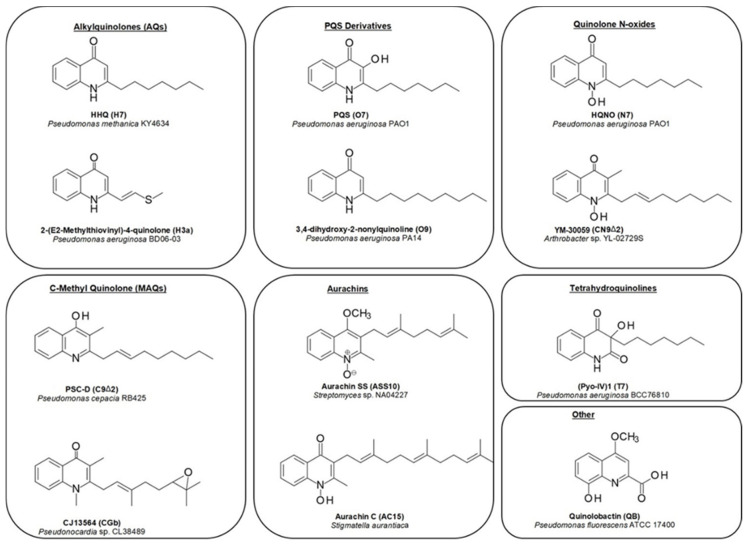
Chemical structures of selected quinolones and producing organisms.

The central structural motif in the alkylquinolones is the 4-quinoline core, an aromatic nitrogen-containing heterocyclic compound that can participate in both electrophilic and nucleophilic substitution reactions (Figure 1). The quinoline scaffold commonly exists in various natural products, exhibiting a broad range of biological activities [15]. In the context of the alkylquinolones, the quinolone nucleus forms the core around which the diverse substituents are arranged. Most of these molecules have alkyl (both saturated and unsaturated) substitution at position 2, though substitution at the quinolone nitrogen atom and C-3 positions are also relatively common [1]. Many of the quinolones exist in equilibrium between the 4-quinolone and 4-hydroxy-quinoline tautomeric forms [16]; the predominance of one over the other is determined largely by the pH [17,18] (Figure 1). Heeb et al. described a nomenclature based on structural predominance at physiological pH [19]. For the purposes of this review, we have represented the structures in the quinolone form wherever possible. The bacterial quinolones discussed in this review can be divided into six major categories, some of which are widely distributed, whilst others are found in only a few rare microbial strains (Figure 2).

The first of these are the classical alkyl-4-quinolones, also known as the hydroxy-alkylquinolones (HAQs) characterized by HHQ and its derivatives, which are substituted solely at the 2-position. The side-chains are most often linear saturated or monounsaturated alkyl chains. While these are most strongly associated with the *Pseudomonas* genus, examples have also been reported from the *Alteromonas*, *Pseudoalteromonas*, and *Burkholderia* genera (Table A1). By far the most widely distributed of these compounds is HHQ itself, but numerous other examples have been elucidated over the years. Alkylquinolones with 1, and 4-11-carbon linear chains have been isolated and fully characterized, though MS-based studies have detected the presence of quinolone derivatives with 1–13 carbon side-chains [20,21,22]. Alkylquinolones with odd-numbered numbers of carbons are more commonly found than those with even numbers of carbon atoms, with seven-carbon and nine-carbon examples being particularly prominent; this may reflect their biosynthesis [23]. More recently, several branched chain examples have been described from *Pseudomonas* (**H7a**, **H8b**) [24] and *Pseudoalteromonas* (**H5a**, **H6a**) strains, respectively [22]. Numerous alkylquinolones with unsaturated alkyl chains have also been described (Figure 2), the best known and most widely distributed of which is 2-(Δ1′-nonenyl-)-4-quinolone (**H9Δ1**), one of the originally reported Pyo series of compounds [6]. The position of the double bond can vary, with quinolones being reported with double bonds at the 1, 2, 3, and 4 positions (Table A1). While the majority of alkylquinolones reported have simple saturated or unsaturated hydrocarbon side chains, a handful of quinolones have been reported with more complex substituents. These include one example containing a cyclopropyl ring in the side chain (**H12a**), first reported from a *P. aeruginosa* strain [25], and sulfide- and benzyl- substituted quinolones (**H3a**, **H7b**), from a Chinese *P. aeruginosa* isolate [24].

The second class are the Pseudomonas Quinolone Signal (PQS) and its derivatives, which are hydroxylated at the 3 position. PQS itself (**O7**), which possesses a seven-carbon alkyl chain at the 2-position, was initially reported from a *P. aeruginosa* strain in 1959, long before its importance as a quorum sensing agent was known [26]. Since then, only a single additional analogue has been isolated, the nonyl-substituted **O9** from a *Streptomyces* species [27], however, other hydroxylated derivatives have been detected using mass spectrometry in several *Pseudomonas* strains [21,28].

Next are the alkyl-4-quinolone *N*-oxides (AQNOs), which attracted much of the early attention on the quinolones due to their significant antibacterial activity. The *N*-oxides also exist as tautomers (hydroxylamine and *N*-oxide forms). The most commonly encountered examples are the heptyl and nonyl-substituted derivatives, though eight and eleven-carbon analogues have also been reported, along with small suite of unsaturated derivatives (Table A1). To date, bacterial quinolone *N*-oxides have only been isolated from members of the *Pseudomonas* genus, with only one exception: a C-3 methylated *N*-oxide (**CN9Δ2**) produced by an *Arthrobacter* species [29]. However, a range of unsaturated derivatives have been detected by MS in *Burkholderia* strains [30].

**Figure 3:**
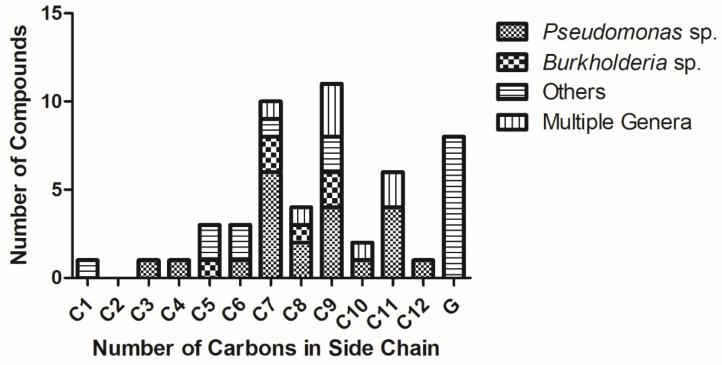
Number of individual isolated alkylquinolones (from all classes) reported vs number of carbons in side-chain (x-axis) from different genera. G = geranyl-derived side chains.

While the majority of quinolones are alkylated solely at the 2-position, a small number are also alkylated at C-3. The most common of these are a group of quinolones bearing a methyl group at the 3 position, known as hydroxy-methyl-alkylquinolines (HMAQs) or methylalkylquinolones (MAQs), which are widely distributed in *Burkholderia* species [31]. The first example, 2-(2-heptenyl)-3-methyl-4-quinolone (**C7Δ2**), was reported from *Burkholderia pyrrocinia* (originally described as *Pseudomonas pyrrocinia*) in 1967 [32]. Several derivatives have since been discovered: the range of sizes of the C-2 substituents are similar to those for other alkylquinolones, with isolated examples incorporating hydrocarbon chains between 5 and 9 carbon atoms long (Table A1). In contrast to the HAQs, all unsaturated HMAQs reported thus far from the *Burkholderia* genus possess unsaturation exclusively at the 2-position of the side-chain. A number of C-3 methylated *N*-oxides have also been detected by metabolic profiling methods (see Section 2), though only one has been isolated and fully characterized [29]. Overall, across all of the quinolone sub-classes substituted at the 2-position, it can be seen that seven and nine-carbon side-chains are the most common (Figure 3). Special note should be made of an intriguing subclass of the alkylquinolones: those containing prenylated side-chains. In a report by Dekker et al. a suite of quinolones (**HG**-**HGc** and **CG**-**CGc**) incorporating geranyl-derived side chains were described from a marine *Pseudonocardia* species, half of which were also methylated at the C-3 position [33]. Some of these compounds were also *N*-alkylated, a feature common in the plant quinolones but very rare in bacterial examples.

Another class of structurally distinct prenylated quinolones are the quinolone-type aurachins, which are substituted by isoprenoid chains at the C-3 position (Table A2), and have been reported from *Stigmatella*, *Rhodococcus*, and *Streptomyces* species [34,35,36,37]. These are unique in that they are the only reported bacterial 4-quinolones to possess alkyl chains larger than a methyl group at the C-3 position.

The last class, the tetrahydroquinolines, are included here as they commonly co-occur with the alkylquinolones and share a biosynthetic origin (Table A2). To date, they have only been reported from *Pseudomonas* species [4,8,9,24,25]. The first isolation of 3-*n*-heptyl-3-hydroxy-1,2,3,4-tetra-hydroquinoline-2,4-dione (**T7**) and 3-*n*-nonyl-3-hydroxy-1,2,3,4-tetra-hydroquinoline-2,4-dione (**T9**) from *P. aeruginosa* was reported by Hays in 1945 as part of the original Pyo series of quinolones [4]. Subsequently, Kitamura isolated **T7** from *P. methanica* [38]. Budzikiewicz reported that **T7** and **T9** were formed when *P. aeruginosa* is grown under iron deficiency [9]. MS profiling studies have revealed the presence of additional analogues with alternative side chains [23].

A handful of bacterial quinolones do not fit into any of these classes. The siderophores quinolobactin and thioquinolobactin are produced by several *Pseudomonas fluorescens* strains, especially under conditions of iron limitation [39,40]. The metabolite 2,4-dihydroxyquinoline (DHQ) has been reported from both *Pseudomonas aeruginosa* and *Streptomyces sindenensis* [20,27].

## 3. Metabolic Profiling

In addition to those quinolones that have actually been isolated and characterized, numerous additional derivatives have been detected by MS-based metabolic profiling. One of the first of these studies was conducted by Taylor et al. in 1995, where GCMS profiling of a clinical isolate of *Pseudomonas aeruginosa* revealed the presence of a large number of alkylquinolones (Table A3) [20]. The major components were the saturated quinolones **H7** and **H9**, in addition to a number of unsaturated analogues with 7, 9, and 11 carbon-chains. GC-MS profiling was also used during the course of a biosynthetic study by Brendenbruch et al., which revealed the presence both PQS and AQ derivatives [28]. Lepine et al. used an LCMS-based method for the analysis of a *P. aeruginosa* strain, which revealed several series of quinolone derivatives: including saturated and unsaturated HAQ derivatives, *N*-oxides, and tetrahydroquinolines. Perhaps most significant were the first detections of C-3 hydroxylated quinolones that were not PQS itself. In a 2017 study, Depke et al. used a new MS-based unsupervised clustering method to analyse an extract from *P. aeruginosa* PA14, identifying a wide range of HAQ, AQNOs, and PQS derivatives. Most notable was the first detection of quinolones containing polyunsaturated side-chains, though only for the longer-chain derivatives (C9 and higher) [41]. In a recent study, a rapid LC-MS method was developed for the analysis of both microbial strains and clinical samples: a total of 28 quinolone derivatives were detected [42]. Other genera also produce quinolones: LC-MS analysis of a marine *Pseudoalteromonas* isolate revealed a suite of saturated alkylquinolone derivatives, including two branched-chain derivatives [22].

*Burkholderia* strains have also been studied—in a 2008 report eleven different *Burkholderia* strains were profiled using LCMS, three of which produced a range of medium-chain HMAQs, HAQs, and methyl-alkylquinoline *N*-oxides (MAQNOs), with dramatically different distributions between the three species [31]. In a biosynthetic study on the effects of KynB on quinolone biosynthesis in *Burkholderia pseudomalleii*, LC-MS profiling was carried out, revealing a similar chemical profile to the prior study [43]. A molecular networking study on the effects of the antibiotic trimethoprim on the secondary metabolites of a *Burkholderia thailandensis* isolate revealed a small suite of C-3 methylated and non-methylated alkylquinolones [44]. A 2020 report described the LC-MS analysis of quinolone derivatives in three microbial strains by multiple reaction monitoring (MRM). With the aid of synthetic standards the authors were able to confirm the location of the double bonds in the unsaturated derivatives: all quinolones with an unsaturated side chain produced by the *Burkholderia* strains possessed a double bond at the 2′-position, while those produced by *Pseudomonas* species had double bonds at either the 1′ or 2′ positions [30].

## 4. Biosynthesis

**Figure 4:**
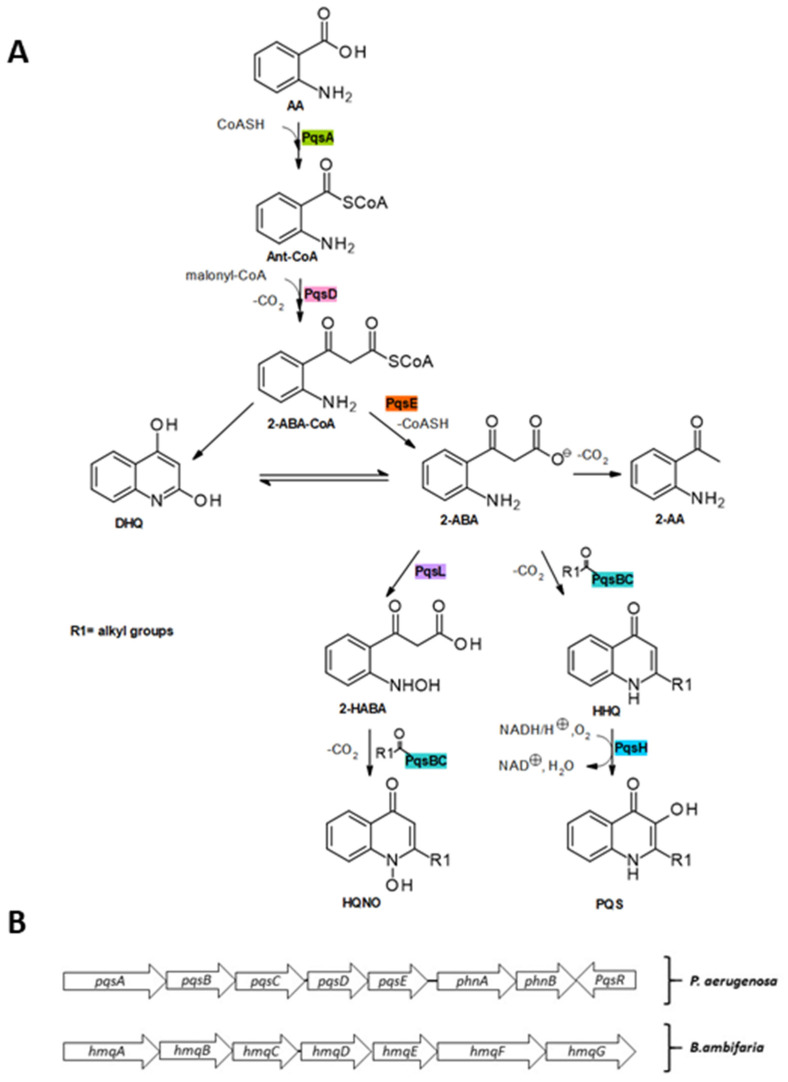
Biosynthesis of 4-quinolones of *Pseudomonas aeruginosa*. (**A**) PqsA catalyzes the activation of AA to Ant-CoA. The decarboxylative coupling reaction of Ant-CoA with malonyl-CoA is catalyzed by PqsD. Hydrolysis of the resulting thioester by PqsE leads to 2-ABA, which is the precursor for the subsequent reactions to DHQ, HQNO, HHQ and PQS. Intermediates and products of the alkylquinolone biosynthetic pathway: AA, anthranilic acid; CoASH, Coenzyme A; Ant-CoA, anthraniloyl-coenzyme; malonyl-CoA; 2-ABA-CoA, 2′-aminobenzoylacetyl-CoA; 2-ABA, 2′-aminobenzoylacetate; DHQ, dihydroxyquinoline; 2-AA, 2′-aminoacetophenone; 2-HABA, 2′-hydroxylaminobenzoylacetate; HQNO, 4-hydroxy-2-heptylquinoline-*N*-oxide; HHQ, 4-hydroxy-2-heptylquinoline; PQS, Pseudomonas Quinolone Signal. (**B**) shows the gene clusters encoding for enzymes responsible for the biosynthesis of quinolones in *Pseudomonas aeruginosa* and *Burkholderia ambifaria.*

Over the past few decades, the biosynthesis of the alkylquinolones has been elucidated [21,45]. The synthesis of HHQ and PQS (Figure 4) is initiated by the coenzyme (CoA) ligase PqsA, which catalyzes the activation of anthranilic acid with ATP/Mg^2+^ to produce the intermediate anthranilyl-AMP and subsequently catalyzes thioesterification with CoA to form anthraniloyl-CoA [46,47,48]. The next step involves the condensation reaction with malonyl-CoA, catalyzed by PqsD, which yields the highly unstable intermediate 2-aminobenzoylacetyl-CoA (2-ABA-CoA) [49]. 

Although 2-ABA-CoA is highly susceptible to spontaneous cyclization to form 2,4-dihydroxyquinoline (DHQ), which has been shown to be fundamental in *P. aeruginosa* pathogenicity [50], in vivo this is counterbalanced by the activity of PqsE, which acts as a 2-ABA-CoA thioesterase to release 2-aminobenzoylacetate (2-ABA) [45]. 2-ABA is another branching point in the pathway: it can undergo decarboxylation to 2-aminoacetophenone (2-AA), a secondary metabolite reported to promote chronic infection phenotypes of *P. aeruginosa* and to modulate the host innate immune response. 2-ABA is transformed into HHQ by the heterodimeric PqsBC bearing an octanoyl chain. Finally, the flavin monooxygenase PqsH oxidizes HHQ into PQS [51]. In addition, 2-ABA could be converted into its hydroxylamine form by the oxidase PqsL and subsequently transformed into 4-hydroxy-2-heptylquinoline-*N*-oxide (HQNO) by the octanoyl-PqsBC complex [52].

## 5. Biological Activity

With their structural diversity, the alkylquinolones also bring a significant number of biological effects. Although most recently the focus has centred on their quorum sensing properties of alkylquinolones, these molecules were initially discovered as antimicrobial agents. Since then, many of these compounds have been isolated for their antibiotic, antifungal and anti-algal activity against human, plant and animal pathogens.

### 5.1. Earlier Discoveries

Based on the works of Bouchard, Emmerich and Löw, Hays et al. described their antibiotic effect and partially characterized the alkylquinolone antibiotics produced by *P. aeruginosa* [4]. They employed serial-dilution assays to test the antibiotic effect of crude extracts, which were later identified as mixtures of 2-alkyl-4-quinolones and their *N*-oxides [4]. These compounds were found to be highly active against Gram-positive bacteria but showed only slight activity against Gram-negative bacteria [4]. It was also shown that the *N*-oxides **N7**–**N9**, **N11** (designated as Pyo-II by Hays et al.) were ten times more potent than the reduced compounds, displaying bactericidal activity at high concentrations and bacteriostatic effects at lower concentrations [4,6,7]. Lightbrown and Jackson later reported that the *N*-oxides are also potent antagonists of streptomycin and dihydrostreptomycin, and inhibit the cytochrome systems of heart muscle and certain bacteria by interfering with the respiratory chain [53,54].

### 5.2. Antibacterial Activity

After the earlier discoveries and introduction of quinolones as front-line antibiotics, a hunt for new natural antimicrobial molecules based on the quinolone scaffold started [16]. The naturally occurring alkylquinolones have proven to be interesting starting point for synthesis of molecules with broad spectrum of activity and less toxicity [16,19]. Wratten et al. reported the isolation of HHQ (**H7**) and its shorter congener, 2-*n*-pentyl-4-quinolone (**H5**/PQ) from a marine *Pseudomonas bromoutalis* isolate. These molecules showed antibiotic activity against both Gram-positive and Gram-negative bacteria including *Staphylococcus aureus,* and the common marine pathogens, *Vibrio harveyi* and *Vibrio anguillarum* [55]. While investigating the ecological and biogeochemical role of a previously-reported alkylquinolone, 2-*n*-pentyl-4-quinolone (**H5**/PQ) from a marine *Alteromonas* sp. SWAT 5, it was determined that **H5** inhibits the growth and motility of several particle-associated bacteria from different phyla including α-Proteobacteria, Bacteroidetes and γ-Proteobacteria. It was also found that **H5** targets DNA synthesis and motility at concentrations as low as 10 µM [56]. In a 1998 report, Debitus and co-workers isolated several quinolone derivatives (**H7**, **H9**, **H9Δ1**, **T7**, **N7**) from a sponge-derived Pseudomonad, with **H7** displaying strong antimicrobial activity [57].

Homma et al. discovered that *Pseudomonas cepacia* RFM25 (subsequently reclassified as *Burkholderia cepacia*) was inhibitory to several soil borne plant pathogens [58]. Later they managed to isolate 2-(2-heptenyl)-3-methyl-4-quinolone (**C7Δ2**), previously isolated by Hashimoto and Hattori, and 2-(2-nonenyl)-3-methyl-4-quinolone (**C9Δ2**) from the same strain [32]. Both molecules displayed similar activity against *Corynebacterium michiganense* [58].

*Helicobacter pylori* infection is one of the most common causes of gastritis and peptic ulcers. While exploring the effects of extracellular substances produced by different bacteria on *H. pylori* it was found that a clinical strain of *P. aeruginosa* inhibited the growth of *H. pylori*. The active fractions were found to contain 2-heptyl-quinolone (**H7**), 2-nonyl-quinolone (**H9**) and their corresponding *N*-oxides. **H7** and **N7** were found to be active against metronidazole and metronidazole resistant strains of *H. pylori* with MIC values of 0.1–0.5 mg/mL in an agar-well plate assay [59]. Another group of bacterial quinolones with antimicrobial activity against *H. pylori* are the geranylated quinolones (**CG**-**CGc**; **HG**-**HGc**) isolated from *Pseudonocardia* spp. CL38489, several of which possessed nanomolar activity. The epoxide derivative 3-dimethyl-2-(6,7-epoxygeranyl)-4-hydroxy-quinolone (**CGb**) was found to be the most potent of all isolates, with a MIC of 0.1 ng/mL [33]. Because of their inactivity against other microbes, and potential of posing no potential harm to normal gut flora, these molecules hold great promise in clinical settings to be used as anti-ulcer agents. One possible explanation for their selective action was postulated to be the selective inhibition of a part of microaerophilic respiratory chain of *H. pylori*.

In 1992 Machan et al. detected a range of quinolone *N*-oxides in a clinical *P. aeruginosa* isolate and identified **N7** as the major active component. Reduction of the *N*-oxide to **H7** with TiCl_3_ significantly reduced the antimicrobial properties against *Staphylococcus aureus* [60]. Exploring the natural product profile of marine sponge-associated *P. aeruginosa* strain, Bultel-Poncé et al. isolated three HAQs (**H9**, **H11**, **H11Δ1**) and a single quinolone *N*-oxide, the latter of which inhibited the growth of *S. aureus*, which was identified as 2-*n*-nonyl-4-quinolone-*N*-oxide (**N9**) [61]. Isolated from *Arthobacter* sp. YL-02729S, in 1996, the methylated *N*-oxide **CN9Δ2** showed moderate activity against Gram-positive bacteria including multiple-drug resistant *S. aureus* and *S. epidermidis* strains, as well as *B. subtilis* [29]. Rattanachuay et al. investigated the anti-*Vibrio* activity and shrimp toxicity of compounds produced by *Pseudomonas* sp. W3 and found that one of the active principles was 2-heptyl-4(1*H*) quinolone (**H7**) [62]. Fractions containing **H7** showed activity against lethal strains of *Vibrio harveyi* (MIC = 450 μg /mL) and a very low toxicity to shrimp [62]. While tapping the alkylquinolone repository of *Streptomyces sindenensis* OUCMDZ-1368*,* Liao et al. reported the isolation of two new alkylquinolones. The newly discovered 2-methyl-4(1*H*)quinolone (**H1**) and 2-nonyl-3-hydroxy-4(1*H*)quinolone (**O9**), along with the known quinolones **H7**, **H9**, **H9Δ1**, **H11** and **O7** (PQS) showed activity against *Staphylococcus aureus* and *Bacillus subtilis* [27].

In a 2016 report, twelve 2-alkyl-4-quinolones, including four new compounds, were isolated from *P. aeruginosa* BCC76810. Amongst the isolated compounds were several 4-quinolone *N*-oxides, including the newly isolated 2-*n*-octyl-4-quinolone *N*-oxide (**N8**) and 2-((*Z*)-undec-4′-enyl)-4-quinolone *N*-oxide (**N11Δ4**) along with the previously-reported **N7** and **N9**, which displayed activity against *Bacillus cereus*, a major cause of foodborne illness, with IC_50_ values of 6.25–25 µg/mL. 2-*n*-octyl-4-hydroxyquinoline *N*-oxide (**N8**) and **N11Δ4** were also shown to be weakly active against *Mycobacterium tuberculosis* with a MIC of 50 µg/mL [25]. Gram-negative bacteria including *Pseudomonas* and *Burkholderia* sp. secrete outer membrane vesicles (OMVs) that contain small molecules important for interactions with surrounding environment and bacteria. Wang et al. found that OMVs of *Burkholderia thailandensis* contain 2-(2-nonenyl)-3-methyl-4-quinolone (**C9Δ2**) that inhibited both drug-resistant and susceptible strains of *S. aureus* and *Acinetobacter baumannii,* and disrupted MRSA biofilms [63].

Li et al. reported the isolation of two new antimicrobial alkylquinolones 2-(2-heptenyl)-4-quinolone (**H7Δ2**) and 2-(*E*-non-2-enyl)-4-quinolone (**H9Δ2**) along with four known ones (**H7**, **C7**, **C7Δ2**, **C9Δ2**) from rhizobacterium of the genus *Burkholderia.* By conducting disc diffusion assays (10 μg/disc) it was found that all these compounds are active against the bacterial fish ulcer pathogen *Tenacibaculum maritimum* [64]. Piochon et al. synthesized six microbial alkylquinolone natural products: **C7Δ2**, **C8Δ2**, **C9Δ2**, **CN7Δ2**, **CN8Δ2**, and **CN9Δ2** and tested them against various gram-positive and gram-negative bacteria. The methyl-alkylquinolone-*N*-oxides (MAQNOs) were found to be more active and exhibited strong activity against gram-positive bacteria (*B. subtilis, S. aureus, Streptococcus agalactiae, and Paenibacillus peori*), but displayed only weak activity against gram-negative bacteria (*Actinobacillus pleuropneumoniae, Xanthomonas campestris*). For the HMAQs, an increase in chain length were found to increase the activity against *A. pleuropneumoniae, B. subtilis,* and *S. aureus*, but the activity was much weaker in comparison to that of MAQNOs [65].

Later, 4-hydroxy-3-methyl-2(1*H*)-quinolone (**CX1**) was isolated from *Burkholderia* sp. 3Y-MMP [66]. This compound was previously isolated as a plant metabolite from *Isatis tinctoria* (*Brassicaceae)* and displayed anti-tuberculosis activity with an IC_90_ of 6.8 μM [66,67,68]. An extensive isolation report in 2020 described the isolation of six new HAQs, including the highly unusual sulfide and benzyl derivatives **H3a** and **H7b**, in addition to fifteen known AQs, three AQNOs, and the tetrhydroquinoline **T7** [24]. While no significant biological studies were reported in the initial isolation report, a subsequent synthetic investigation revealed that the natural products **H3a**, **H7b** and **H7Δ2** inhibited the growth of *S. aureus*, with the latter reducing growth by over 80% at 1.1 μM. The latter two compounds also reduced the swarming motility of *B. subtilis* [69].

Kunze et al. described the antimicrobial effect of the aurachins, several of which of are isoprenoid quinolone alkaloids characterized by a farnesyl residue, from the myxobacterium *Stigmatella aurantiaca* [34]. Aurachins C (**AC15**) and D (**AD15**) were shown to block NADH oxidation and inhibit the growth of several gram-positive bacteria including *B. subtilis, S. aureus, Arthrobacter aurescens, Brevibacterium ammoniagenes,* and *Corynebacterium fascians*; potentially by interfering with bacterial respiration [34]. Additional antimicrobial aurachins have been reported: Kitagawa et al. isolated aurachin RE (**ARE15**) from *Rhodococcus erythropolis* JCM 6824 which exhibited a broader and more potent activity than aurachin C [34,35]. It was found to inhibit both Gram positive (*B. subtilis, Nocardia pseudosporangifera*, *Streptomyces griseus*) and Gram-negative bacteria (*Sinorhizobium meliloti* and *Deinococcus grandis*). Aurachins Q (**AQ15**) and R (**AR15**) from *Rhodococcus* sp. Acta 2259 displayed low to moderate activity against *S. epidermidis*, *B. subtilis* and *P. acnes,* respectively [34,35,36]. The *O*-methylated aurachin SS (**ASS10**), isolated from a *Streptomyces* strain, possessed reduced antimicrobial activity compared to aurachins C and D, though it is unclear whether this is due to the presence of the methoxy group, or the shortened side-chain [37].

Many researchers have studied the structure–activity relationships of the synthetic quinolone antibiotics [70], but the relationships of the bacterial alkylquinolones have not been as well-established. While it has been commonly observed that the alkylquinolone *N*-oxides (AQNOs) have a broader spectrum of activity than their reduced counterparts it is clear that the length, degree of unsaturation and branching of the alkyl chain also play a role. For example, Szamosvári (2017) reported that the unsaturated compound *trans*-(non-1-enyl)-4-quinolone *N*-oxide exhibited up to 20-fold higher bacterio-static activity against *Staphylococcus* *aureus* strains than the most potent saturated AQNO [71]. Due to the diverse test organisms and methods used in the studies overviewed in this section, it is difficult to establish firm rules for antibacterial activity. Nonetheless, alkylquinolones possessing an unsaturated chain and either *N*-methylation or *N*-oxidation tend to have improved antimicrobial properties. Alkylquinolones with unsaturated carbon chains and methylation at R-3 do tend to possess good anti-bacterial activity, but such structural modifications are not essential for good anti-bacterial activity [32,64]. An unusual example seems to be the geranylated quinolones, which possess potent and selective activity against *H. pylori*, with epoxidation of the geranyl residue (**CGb**) significantly increased the potency of the molecule [33]. For the aurachins, the addition of a double bond at position 4 of the prenylated chain (**AQ15**, **AR15**) results in loss of activity, whereas addition of hydroxy group in the side-chain (**ARE15**) increased the spectrum of activity to a great extent [34,35].

### 5.3. Anti-Algal Activity

Algae cause a great deal of economic losses annually and pose a great threat to humans, plants and wildlife [72,73,74]. 2-undecyl-4-quinolone (**H11**) and 2-undecen-1′-yl-4-quinolone (**H11Δ1**) isolated from *Alteromonas* sp. KNS-16 proved to be highly effective against harmful algal blooms (HAB)-causing algae *Heterosigma akashiwo* and *Cochlodinium polykrikoides*. With an LC_50_ of 0.5 µg/mL, **H11Δ1** proved to be more potent against *H. akashiwo* [75]. Algae also play an important role in marine and freshwater ecosystems and while studying the role of bacterial antibiotics on bacterium-bacterium and bacterium-phytoplankton interactions Long et al. found that **H5** from a marine *Alteromonas* sp. inhibits diatoms *Thalassiosira weissflogii*, *Chaetoceros. simplex*, and *Clavulinopsis fusiformis* and the cyanobacterium *Synechococcus* sp. [56].

### 5.4. Antifungal and Anti-Oomycete Activity

Fungal and oomycete pathogens cause massive economic losses by effecting crops in both temperate and tropical areas. 2-(2-heptenyl)-3-methyl-4-quinolone (**C7Δ2**) and 2-(2-nonenyl)-3-methyl-4-quinolone (**C9Δ2**) from *Pseudomonas cepacia* RFM25 inhibited growth of the common plant pathogen *Pythium ultimum* [58]. Both compounds showed very strong inhibition against *Verticillium dahlia*, moderate inhibition against *Pyricularia oryzae* and *Cochliobolus miyabeanus*, and weak growth inhibition against *Rhizoctonia solani, Fusarium oxysporum* and *Gaeumannomyces graminis* [58]. **C7Δ2** and **C9Δ2** are therefore potential candidates to prevent severity of radish damping-off and wilting in tomato eggplant. A range of HMAQs (**C5**, **C7Δ2**, **C7**, **C9Δ2**, **C9**) obtained from *P. cepacia* PC-II (subsequently renamed *Burkholderia cepacia*) antagonized the pathogenic effect of *Phytophthora capsica*, which is responsible for ‘phytophthora blight’ in red peppers. **C7Δ2** proved to be the most potent, with MIC between 32–128 µg/mL against the oomycetes *Phytophthora capsici, Pythium ultimum* and the fungi *Fusarium oxysporum, and Rhizoctonia solani.* Furthermore, coating celite containing **C7Δ2** onto red pepper seeds enhanced their growth [76].

During a co-culture experiment it was found that *P. aeruginosa* exhibited a fungicidal effect on *Cryptococcus neoformans*. On further investigation it was discovered that the anti-fungal action was due to pyocyanin and the alkylquinolones PQS (**O7**) and HHQ (**H7**) produced in the co-culture [77].The quinolones **H1**, **H7**, **H9**, **H9Δ1**, **H11**, **O7** and **O9** from an alkali-tolerant *Streptomyces sindenensis* were found to inhibit the growth of *C. albicans* one of the most prevalent causes of opportunistic fungal infections in humans [27]. Aurachins C and D were also shown to inhibit the growth of the yeasts *Saccharomyces cerevisiae* and *Debaryomyces hansenii* [34]. A small suite of quinolones (**C7**, **C7Δ2**, **H9Δ2**, **C9Δ2**), isolated from a *Burkholderia* species, displayed antifungal activity against the fungal pathogens *Rhizopus oryzae* and *Trichophyton rubrum* [64]. Mossialos et al. discovered the iron-chelating properties of quinolobactin, isolated from a *Pseudomonas fluorescens* ATCC 17400 strain [39]. Although quinolobactin (**QB**), originally reported along with thioquinolobactin in 1980 [78], did not show any significant activity besides iron chelation it was later demonstrated that quinolobactin is a produced by the hydrolysis of the unstable but highly active 8-hydroxy-4-methoxy-2-quinoline thiocarboxylic acid (thioquinolobactin). Thioquinolobactin (**TQB**) not only possesses iron chelation properties but also significantly inhibits the growth of *Pythium debaryanum, Rhizoctonia solani* and *Sclerotinia sepivorium* [40]. Later Kilani-Feki et al. also isolated **C7** and **C7Δ2** from *B. cepacia* and demonstrated their activity against common food pathogen *Aspergillus niger* [79,80]. **C9Δ2** from outer membrane vesicles (OMVs) of *B. thailandensis* displayed anti-fungal activity at 100 µM against *C. albicans* and *Cryptococcus neoformans* [63]. Microbial alkylquinolones **C7Δ2**, **C8Δ2**, **C9Δ2,** obtained by employing Conrad−Limpach approach and Suzuki−Miyaura coupling reactions, showed moderate activity against *C. neoformans* [65]. Overall, for anti-fungal and anti-oocyte activity of the alkylquinolones, methylation of position-3 and the presence of an unsaturated alkyl chain at C-2 are strong predictors of antifungal activity.

### 5.5. Quorum Sensing/Biofilm Formation

The ability of bacteria to communicate and act as a community for collective tasks was underestimated for many years. It was believed that these organisms act on the principle of ‘every cell for itself’ but later it was realized that the bacterial cells communicate by a phenomenon called quorum sensing, or auto-induction [81]. The concentrations of auto-inducer molecules released by a single bacterium are insufficient to bring about behavioural and metabolic changes. Thus, this phenomenon occurs in a cell density-dependent manner, where the bacteria must reach a critical mass necessary for collective action, to activate or supress target genes by releasing auto-inducers [82]. The collective action of such molecules and the regulation of an array of genes help these bacteria move to a more friendly environment (with better nutrients), adapt a new growth strategy, or aid in protection from harsh/deleterious environments by biofilm formation [82].

After the discovery of the quorum sensing activity of 2-heptyl-3-hydroxy-4-quinolone, termed Pseudomonas quinolone signal (PQS) by Pesci et al. in 1999, much investigation has been done on the exact nature of the mechanism by which these small molecules interact with each other and the target genes [12]. Further *P. aeruginosa* alkylquinolones have been found to be involved in quorum sensing in *Pseudomonas putida* and *Burkholderia* sp. as well [83]. Two distinct structural classes of alkylquinolones have been found to be involved in quorum sensing: the hydroxy-alkylquinolines (HAQs) and hydroxy-methyl-alkylquinolones (HMAQs) [31]. HAQs involved in quorum sensing include 2-heptyl-3-hydroxy-4-quinolone (**O7**/PQS), its precursor 4-hydroxy-2-heptyl-quinolone (**H7**/HHQ), and the related compound 2-nonyl-4-hydroxyquinoline (**H9**/NHQ) [84].

At a transcriptional level, the biosynthesis of alkylquinolones is controlled by PqsR, which activates the expression of *pqsABCDE* and *phnAB* operons in *P. aeruginosa* [85]. A homologue of the pqsABCDE operon named *hmqABCDEFG* has been found in *Burkholderia* spp. (Figure 4) [31,86]. Some of these autoinducers i.e., PQS and HHQ, regulate their own expression by binding to PqsR itself [87]. It has been revealed that HHQ induces a conformational change in PqsR, as binding of PqsR to the *pqsA* promoter in vitro is enhanced by HHQ, although not as much as with PQS [88]. Other AQs such as 2-nonyl-4-quinolone (**H9**) can also activate PqsR and as such could potentially be considered as autoinducers, although not as potent as PQS [84]. Alkylquinolones are highly lipophilic molecules, which might hinder their ability to act as effective signaling molecules, however, several of the biochemical changes they induce work to minimize this problem. PQS increases the production of rhamnolipids, which increases the solubility of this lipophilic molecule within aqueous solutions [89]. Another induced change that may help to overcome solubility issues is via promoting the biogenesis of outer membrane vesicles (OMVs) that package the highly lipophilic PQS for transport within the population [90]. It has been found that the third hydroxy position is absolutely critical for the formations of OMVs and is the reason why PQS and not HHQ can stimulate OMV formation [91]. The kinetics of both HAQs and HMAQs show that these molecules start to accumulate near the end of the log phase of growth of the respective bacteria [31].

Besides regulating adaptation responses and signal integration, these alkylquinolone derivatives are primarily involved in the regulation of various genes responsible for virulence. The virulence factors mainly regulated by these auto-inducers include elastase, pyocyanin, hydrogen cyanide, rhamnolipids and LecA lectin [86,92]. The production of these molecules gives a competitive advantage to the bacterium producing them [93]. This advantage is demonstrated by modulating the swarming motility, growth repression by depriving the rival bacteria of iron i.e., iron chelation, cytotoxicity via production of reactive oxygen species, and regulating the production of antimicrobial AQs like 4-hydroxy-2-heptylquinoline-*N*-oxide (HQNO) (**N7**) [91,94,95,96,97,98]. Although not an actual quorum sensing molecule itself, HQNO has been found to induce antibiotic tolerance by triggering programmed cell death. The DNA released due to cell death can induce biofilm formation making the bacteria resistant to antibiotics. This antibiotic tolerance can be triggered not only in the species producing the molecule i.e., *P. aeruginosa,* but also in the bacteria in the surrounding environment e.g., *S. aureus* [99,100]. It should be noted that the growth repression function is distinct from bacteriostatic or bactericidal effect of antibiotics [94]. Biofilm formation is a complex phenomenon, the mechanism of which is not fully understood, but it has been inferred that alkylquinolone quorum sensing molecules promote biofilm formation via LecA activation and/or formation of extracellular DNA and may also promote or inhibit the biofilm formation in other bacteria [97,101,102].

**Figure 5:**
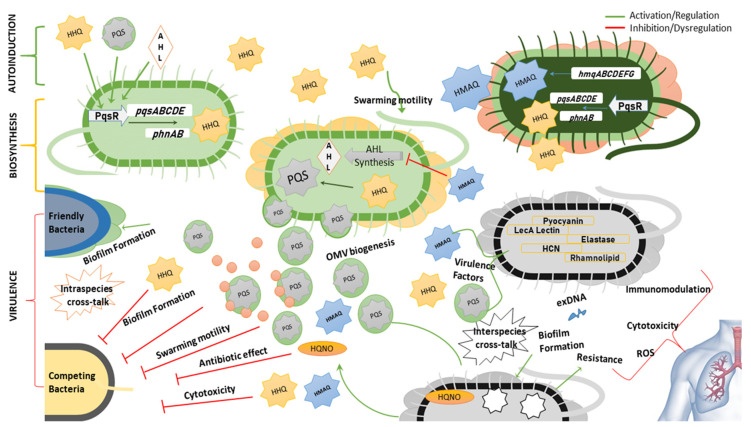
Summary of links between biosynthesis, auto-induction and virulence mediated by alkylquinolone quorum sensing molecules. Abbreviations: PQS—Pseudomonas Quinolone Signal, HHQ—4-hydroxy-2-heptyl-quinolone, HMAQ—hydroxy-methyl-alkylquinolones, AHL—acyl-homoserine lactones, HQNO—4-hydroxy-2-heptylquinoline-*N*-oxide, HCN—hydrogen cyanide, OMV—outer membrane vesicles, ROS—reactive oxygen species, exDNA—extracellular DNA.

Reen et al. discovered that the C-3 position is crucial for the wide range of activities including production of virulence factors and biofilm formation exhibited by PQS and HHQ. Absence or substitution of this position with halogens or an NH group results in loss of activity in modulating inter-species and intra-species behaviour [103]. It has also been observed that PQS not only acts by transcriptional regulation within the cells but may also interact directly with the proteins e.g PQS binds with MexG (RND-type efflux pump) and MgtA (Mg^2+^ transporter) [104]. It has also been demonstrated that the two quorum sensing systems in *Pseudomonas* and *Burkholderia* spp. work interdependently. The acyl-homoserine lactones (AHLs) regulate the expression of HAQs via LasR and RhlR which bind to PqsR [105]. On the other hand, hydroxymethyl-alkylquinolines (HMAQs) found in *Burkholderia* spp. regulate the expression of AHLs and it has been demonstrated that the methyl group of HMAQs is essential for this function [31]. The role of quinolones in quorum sensing, virulence, and interspecies interactions is summarized in Figure 5.

### 5.6. Antimalarial Activity

The isolation of quinine from *Cinchona* bark was the starting point of modern-day quinolones used in clinical settings. Due to the structural similarities between quinine and the quinolones, researchers have also explored the antimalarial potential of alkylquinolones. 2-undecyl-4-quinolone (**H11**), 2-undecen-1′-yl-4-quinolone (**H11Δ1**) and 2-nonyl-4-quinolone (**H9**), isolated from a sponge-associated *Pseudomonas* sp. showed activity against *Plasmodium falciparum* at ID_50_ of 1.0, 3.8 and 4.8 µg/mL respectively [61]. Supong et al. reported the isolation of antimalarial 4-alkylquinolones with IC_50_ values in a range of 0.25–2.07 µg/mL. The compounds were isolated from *P. aeruginosa* BCC76810 and included quinolones **H7**-**9**, **H9Δ1**, **H11**, **H11Δ4**, **N7**, **N8**, **N9**, **N11** and **N11Δ4**, of which **H11** was the most active, with an IC_50_ of 0.25 µg/mL [25]. Another antimalarial alkylquinolone, 2-(6-methyl)-heptyl-4-quinolone, (**H8a**) was recently isolated from *P. aeruginosa* BD06-03 [66]. This compound has been previously shown to inhibit the growth *P. falciparum*, with an IC_50_ of 583 nM [106]. Based on the above data, it suggests that a longer chain length increases the antimalarial potency of the alkylquinolones.

### 5.7. Miscellaneous Activities

Alkylquinolones have mostly been analyzed for their antimicrobial and quorum sensing abilities. But these unique quinolones have proven to be very diverse compounds in terms of their activity despite of their limited structural diversity. 2-nonyl-4-quinolone (**H9**), one of the oldest alkylquinolones known from *P. aeruginosa* was isolated from Brazilian shrub *Raulinoa echinata* by Biavatti et al. in 2002 and showed moderate antitrypanosomal activity against *T. cruzi* with an IC_50_ 100.9 µg/mL [6,107]. 2-undecyl-4-quinolone isolated from *Pseudomonas* sp. 1531 E7 displayed antiviral against HIV at an ID_50_ of 10^−3^ μg/mL.

2-*n*-Heptyl-4-hydroxy-quinoline-*N*-oxide (**N7**), 2-*n*-heptyl-4-quinolone (**H7**), 3-*n*-heptyl-3-hydroxy-1,2,3,4-tetrahydroquinoline-2,4-dione (**T7**) were analyzed for their anti-asthma activity via 5-lipooxygenase. Although all these compounds showed 5-lipooxygenase inhibition activity, **N7** proved to be the most potent and selective inhibitor (IC_50_ = 1.5 × 10^−7^ M) [38]. Later **N7** was also shown to significantly suppress the antigen-induced bronchoconstriction in guinea pigs and inhibition of histamine release [108]. 2-*n*-heptyl-4-quinolone (**H7**) from marine *Pseudoalteromonas* sp. M2 was investigated for its anti-inflammatory activity and proved to be a very promising candidate for neuro-inflammatory disorders, owing to its ability to inhibit NO, ROS production and the expression of iNOS and COX-2 [109,110]. The same compound (**H7**) proved to be the most potent inhibitor of melanin synthesis when tested for anti-melanogenic activity. A range of AQs also showed strong activity against Hep C virus with an IC_50_ of 1.4 ± 0.2 μg/mL and no cytotoxicity. This activity was stronger than that of ribavirin and an investigation into the mechanism of action revealed that **H9** acts against HCV by inhibiting the viral entry and replication [111]. **H9** has also been found to possess iron-chelation properties [112]. Aurachins, including **AC12** and **AD12** from *Rhodococcus* sp. Acta 2259 showed weak inhibition of glycogen-synthase-kinase 3β (GSK-3β) which could lead to exploration of these molecules for treatment of neurodegenerative and other disorders caused by perturbation of GSK-3β [36].

Insulin like growth factor-1 (IGF-1) is a key player in human growth and development and its perturbation can cause severe diseases including cancer, acromegaly, diabetes, thyroid eye disease, acne and psoriasis [113]. (E)-3-methyl-2-(2-octenyl)-4- quinolone (**C8Δ2**) isolated from *Burkholderia* sp. QN15488 induces death in 32D/GR15 cells and many cancer cells including MCF-7 and HeLa cells via both IGF-1 dependent and independent manner [114]. Kamigiri et al. determined the cytotoxicity of 1-hydroxy-2-(non-2-enyl)-3-methyl-4-quinolone (**CN9Δ2**) against HeLa S3 cells in vitro with an IC_50_ of 0.59 µg/mL [29]. With IC_50_ < 2 µg/mL the alkylquinolone 2-nonyl-4-quinolone *N*-oxide (**N9**) showed high cytotoxicity toward human epidermal carcinoma KB cell line along with 2-undecen-1′-yl-4-quinolone (**H11Δ1**) which was weakly cytotoxic [61]. 2-Alkylquinolones **H1**, **H7**, **H9**, **H9a**, **H11**, and 2-alkylquinolone *N*-oxides **O7** and **O9**, derived from *Streptomyces sindenensis* showed cytotoxicity against a A549 cell line [27]. All the alkylquinolones isolated from *P. aeruginosa* BCC76810 by Supong et al. showed moderate cytotoxicity against cancer cells and were weakly toxic to normal cells [25]. Antioxidant properties have also been found in the alkylquinolones: the *N*-oxide derivatives **N7**-**N9** and **N11Δ4** displayed antioxidant activity via the DPPH assay [25].

## 6. Conclusions

**Figure 6:**
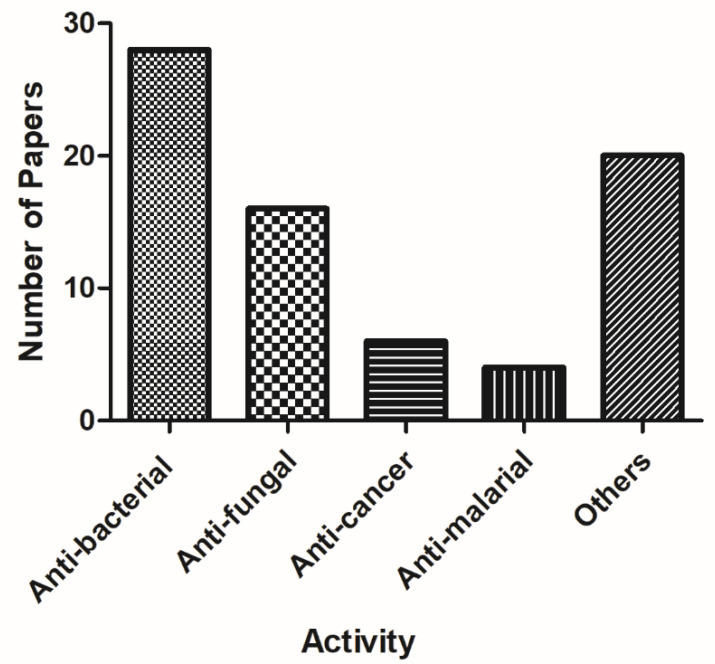
Number of papers reporting various activities of bacterial alkylquinolones.

Overall, the alkylquinolones are a chemically and biologically diverse class of compounds. Initially discovered from the *Pseudomonas* genus, recent studies have shown that these compounds are distributed amongst several genera, including not only *Burkholderia*, but also more distantly related genera such as *Streptomyces* and *Rhodococcus*. Likewise, while many of the earlier-discovered alkylquinolones incorporated only linear C-2 side chains, more recent isolation studies have greatly diversified the number of structural motifs encountered in this structure class. While the majority of studies have focused on their antimicrobial properties (Table A1 in Appendix A, Figure 6), it is clear that these compounds have potential as anti-fungal, anti-malarial, and anti-inflammatory agents as well, and that future investigators would do well to broaden the scope of biological activities under investigation.

## Appendix A

**Table A1:** Alkyl-4-quinolones isolated from bacteria.

						Isolated from				Biological Activity			
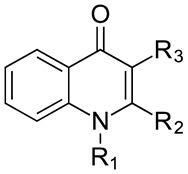
Number	R_1_	R_2_	R_3_	Formula	Year 1st Isolated	*Pseudomonas*	*Burkholderia*	Other	AB	AF	AC	AM	Other
*Simple Alkylquinolones*													
H1	H	CH_3_	H	C_10_H_9_NO	2015 [27]			[27]					
H3a	H		H	C_12_H_11_NOS	2020 [24]	[24]			[69]				
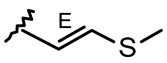
H4	H		H	C_13_H_15_NO	2016 [22]			[22]					[22]
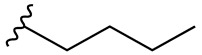
H5 (PQ/ pseudane-V)	H		H	C_14_H_17_NO	1977 [55]			[22,55,56]	[55,56]				[22,56]
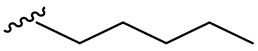
H5a	H		H	C_14_H_17_NO	2016 [22]			[22]					[22]
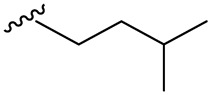
H6 (pseudane-VI)	H		H	C_15_H_19_NO	2016 [22]			[22]					[22]
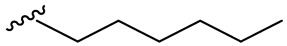
H6a	H		H	C_15_H_19_NO	2016 [22]			[22]					[22]
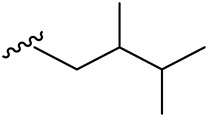
H6Δ2b	H		H	C_15_H_17_NO	2020 [24]	[24]							
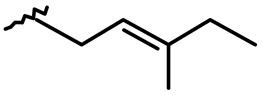
H7 (HHQ/ pseudane-VII/Pyo-1b)	H		H	C_16_H_21_NO	1945 [4]	[4,10,24,25,38,57,59,60,62,112,115,116]	[63,64]	[22,27,55,56,109]	[4,6,27,55,57,59,60,62,64]	[77]	[22,25,106]	[106]	[22,25,38,109,110]
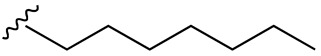
H7a	H		H	C_16_H_21_NO	2020 [24]	[24]							
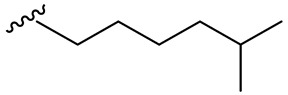
H7Δ1	H		H	C_16_H_19_NO	1976 [117]	[10,24,116,118]							
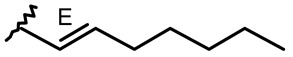
H7Δ2	H		H	C_16_H_19_NO	2018 [64]	[24]	[64]		[64,69]				
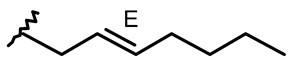
H7b	H		H	C_16_H_13_NO	2020 [24]	[24]			[69]				
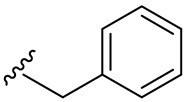
H8 (pseudane-VIII)	H		H	C_17_H_23_NO	2013 [63]	[24,25,63]		[22]			[22,25,106]	[25,106]	
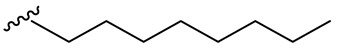
H8D1	H		H	C_17_H_21_NO	2020 [24]	[24]							
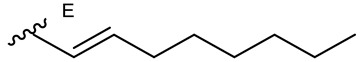
H8a	H		H	C_17_H_23_NO	2020 [24]	[24]					[106]	[106]	
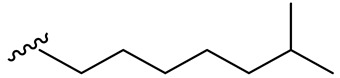
H9 (HNQ/ pseudane-IX/ Pyo-Ic)	H		H	C_18_H_25_NO	1945 [4]	[4,10,24,25,57,59,60,61,63,112,116]		[22,27]	[4,6,21,27,59]		[25]	[25,61]	[107,111,112]
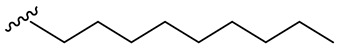
H9Δ1 (Pyo-III)	H		H	C_18_H_23_NO	1945 [4]	[4,7,10,24,25,57,63,116]		[27]	[4,6,27]		[25]	[25]	
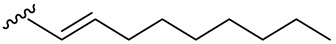
H9Δ2	H		H	C_18_H_23_NO	2018 [64]	[24]	[63,64]		[63,64]				
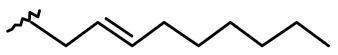
H9Δ4	H		H	C_18_H_23_NO	2020 [24]	[24]							
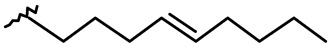
H9a	H		H	C_18_H_25_NO_2_	2020 [24]	[24]							
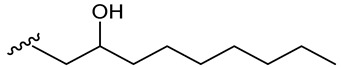
H10 (pseudane-X)	H		H	C_19_H_27_NO	2016 [22]	[22,24]		[22]					[22]
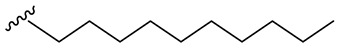
H10Δ1	H		H	C_19_H_25_NO	2020 [24]	[24,25,61]							
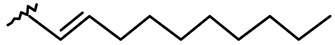
H11 (pseudane-XI)	H		H	C_20_H_29_NO	1999 [61]	[24,25,61]		[22,27,75]			[22,25,61]	[25,61]	[61,75]
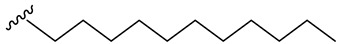
H11Δ1	H		H	C_20_H_27_NO	1999 [61]	[24,61]		[75]			[25]	[25]	[75]
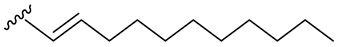
H11Δ3 (Pyo-V)	H		H	C_20_H_27_NO	1979 [10]	[10]							
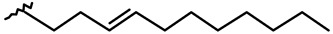
H11Δ4	H		H	C_20_H_27_NO	2016 [25]	[24,25]							
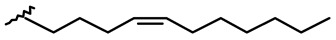
H12a	H		H	C_20_H_27_NO	2016 [25]	[24,25]							
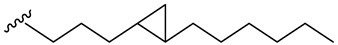
HG (CJ 13565)	H		H	C_19_H_23_NO	1998 [33]			[33]	[33]				
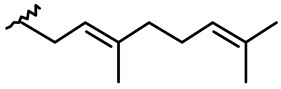
HGa (CJ 13566)	CH_3_		H	C_20_H_25_NO	1998 [33]			[33]	[33]				
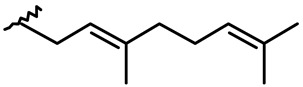
HGb (CJ 13567)	CH_3_		H	C_20_H_25_NO_2_	1998 [33]			[33]	[33]				
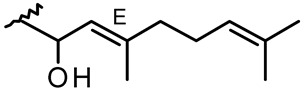
HGc (CJ 13568)	CH_3_		H	C_20_H_25_NO_2_	1998 [33]			[33]	[33]				
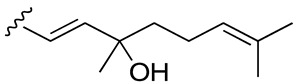
*Pseudomonas Quinolone Signal and derivatives*													
O7 (PQS)	H		OH	C_16_H_21_NO_2_	1959 [26]	[12,21,23,26,118,119]		[27]	[27]	[77]			[12]
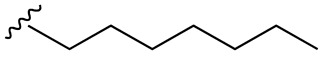
O9	H		OH	C_18_H_25_NO_2_	2015 [27]	[21,119]		[27]	[27]				
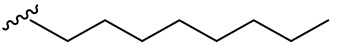
*Alkylquinolone N-oxides*													
N7 (Pyo II, HQNO)	OH		H	C_16_H_21_NO_2_	1945 [4]	[4,7,24,25,38,53,57,59,60,99,120]			[4,7,25,53,59,60,120]		[25]	[25]	[25,38,53,54,97,108]
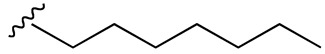
N8	OH		H	C_17_H_23_NO_2_	1956 [7]	[7,21,23,25]			[25]		[25]	[25]	[25]
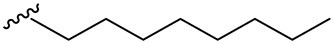
N9 (Pyo II, NQNO)	OH		H	C_18_H_25_NO_2_	1945 [4]	[4,7,24,25,61]			[4,7,25,61,71]		[25,61]	[25]	[25,54,71,102]
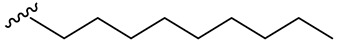
N11	OH		H	C_20_H_29_NO_2_	1945 [4]	[4,7]			[4,7]				[54]
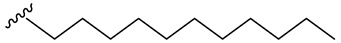
N11Δ4	OH		H	C_20_H_27_NO_2_	2016 [25]	[24,25]			[25]		[25]	[25]	[25]
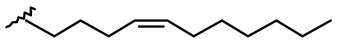
*C-3 methyl Alkylquinolones*													
CX1	H	OH	CH_3_	C_9_H_9_NO_2_	2020 [66]		[66]						[66,67]
C5 (PSC-A)	H		CH_3_	C_15_H_19_NO	1996 [76]		[76]						
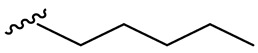
C7 (PSC-C)	H		CH_3_	C_17_H_23_NO	1996 [76]		[64,76,79,80]		[64]	[64,79,80]			
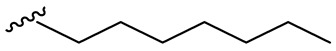
C7Δ2 (PSC-B)	H		CH_3_	C_17_H_21_NO	1967 [32]		[32,58,64,76,79,80,121]		[58,64,65]	[58,64,65,76,79,80]			
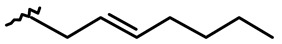
C8Δ2	H		CH_3_	C_18_H_23_NO	2007 [114]		[114]		[65]	[65]	[114]		
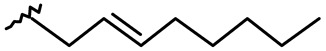
C9 (PSC-E)	H		CH_3_	C_19_H_27_NO	1996 [76]		[31,76]						
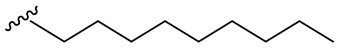
C9Δ2 (PSC-D)	H		CH_3_	C_19_H_25_NO	1989 [58]		[58,63,64,76]		[58,63,64,65]	[58,64,65]			
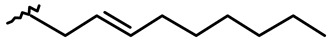
CN9Δ2	OH		CH_3_	C_19_H_25_NO_2_	1996 [29]			[29]	[29,30,65]		[29]		
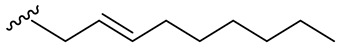
CG (CJ 13136)	H		CH_3_	C_20_H_25_NO	1998 [33]			[33]	[33]				
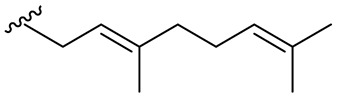
CGa (CJ 13217)	CH_3_		CH_3_	C_21_H_27_NO	1998 [33]			[33]	[33]				
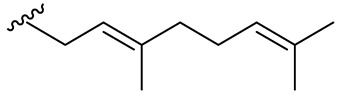
CGb (CJ 13564)	CH_3_		CH_3_	C_20_H_25_NO_2_	1998 [33]			[33]	[33]				
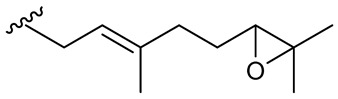
CGc (CJ 13536)	CH_2_SMe		CH_3_	C_22_H_29_NOS	1998 [33]			[33]	[33]				
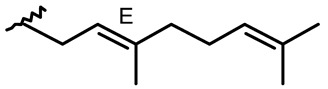

AB—Anti-bacterial, AF—Anti-fungal and anti-oomycete, AC—Anti-cancer and cytotoxic, AM—Antimalarial, Others—Anti-algal, Antioxidant, Auto induction etc.

**Table A2:** Bacterial alkylquinolones with alkyl substitution at position 3 and quinolobactins.

				Isolated from				Biological Activity			
Number	Structures	Formula	Year 1st Isolated	*Pseudo.*	*Burk.*	Other	AB	AF	AC	AM	Others
T7 (Pyo-IV)1		C_16_H_21_NO_3_	1945 [4]	[4,8,24,25,38,57]							[25,38]
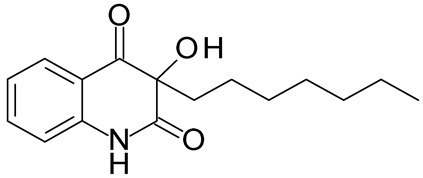
T9 (Pyo-IV)2		C_18_H_25_NO_3_	1945 [4]	[4,8,24,25]							
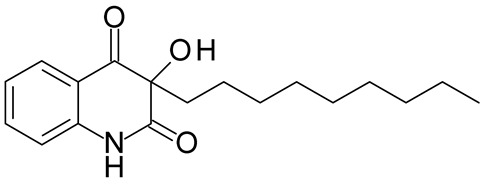
AC15 (Aurachin C)		C_25_H_33_NO_2_	1987 [34]			[34,36,37]	[34,36,37]	[34]			[36]
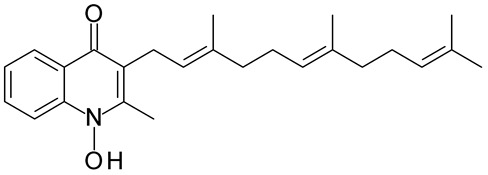
AD15 (Aurachin D)		C_25_H_33_NO_2_	1987 [34]			[34,36,37]	[34,37]	[34]			
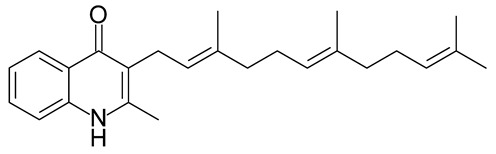
AQ15 (Aurachin Q)		C_25_H_3_NO	2010 [36]			[36]					
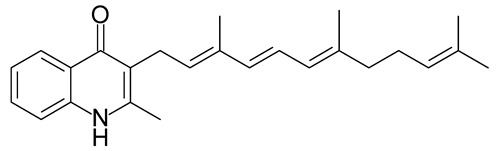
AR15 (Aurachin R)		C_25_H_31_NO_2_	2010 [36]			[36]	[36]				[36]
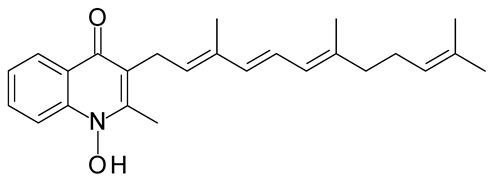
ARE15 (Aurachin RE)		C_25_H_33_NO_3_	2008 [35]			[35]	[35]				
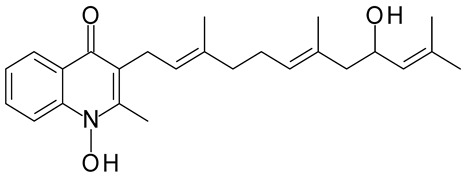
ASS10 (Aurachin SS)		C_21_H_27_NO_2_	2017 [37]			[37]	[37]				
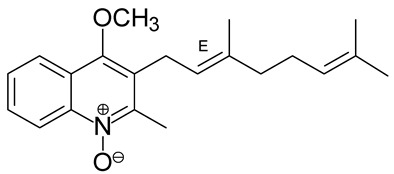
QB (Quinolobactin)		C_11_H_9_NO_4_	1980 [78]	[39,40,78]							
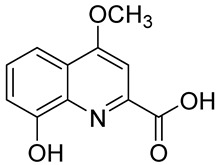
TQB (Thioquinolobactin)		C_11_H_9_NO_3_S	2007 [40]	[40,78]				[40]			
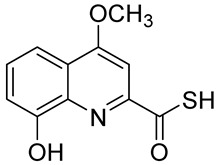

AB—Anti-bacterial, AF—Anti-fungal and anti-oomycete, AC—Anti-cancer and cytotoxic, AM—Antimalarial, Others—Anti-algal, Antioxidant, Auto induction etc.

**Table A3:** Side-chain lengths of alkyl-4-quinolone derivatives detected during MS-based metabolic profiling of microbial extracts.

Reference	Species	AQs (Satd)	AQs (Unsatd)	AQNOs (Satd)	AQNOs (Unsatd)	PQS Derivatives (Satd)	PQS Derivatives (Unsatd)	Tetrahydro-Quinolines (Satd)	Tetrahydro-Quinolines (Unsatd)	MAQs (Satd)	MAQs (Unsatd)	MAQ-NOs (Satd)	MAQ-NOs (Unsatd)
Taylor 1995 [20]	*P. aeruginosa*	C_1_–C_13_	C_2_–C_12_	-	-	-	-	-	-	-	-	-	-
Lepine 2004 [23]	*P. aeruginosa*	C_5_–C_13_	C_5_–C_13_	C_5_–C_11_	C_7_–C_12_	C_7_–C_9_	-	C_5_–C_9_, C_11_	C_5_, C_7_, C_9_	-	-	-	-
Bredenbruch 2005 [28]	*P. aeruginosa*	C_7_, C_9_, C_11_	-	-	-	C_7_, C_9_	-	-	-	-	-	-	-
Depke 2017 [41]	*P. aeruginosa*	C_1_–C_13_, C_15_	C_5_–C_13_, C_15_, C_17_	C_1_–C_3_, C_5_–C_11_, C_13_	C_3_, C_5_, C_7_–C_13_	C_7_–C_9_	C_7_, C_9_–C_11_	-	-	-	-	-	-
Szamosvári 2017 [71]	*P. aeruginosa* PAO1	-	-	C_3_, C_5_, C_7_, C_9_	C_9_	-	-	-	-	-	-	-	-
Brewer 2020 [42]	*P aeruginosa*	C_5_–C_11_	C_7_–C_11_	C_5_–C_9_, C_11_	C_7_–C_9_	C_5_–C_9_	C_7_, C_9_	-	-	-	-	-	-
Szamosvári 2020 [30]	*P. aeruginosa* *PAO1*	C_6_–C_9_, C_11_	C_6_, C_9_, C_11_	C_5_–C_9_	C_7_–C_10_	-	-	-	-	-	-	-	-
Szamosvári 2020 [30]	*P. aeruginosa* PA14	C_6_–C_9_, C_11_	C_6_, C_9_, C_11_	C_5_–C_9_	C_7_–C_10_	-	-	-	-	-	-	-	-
Szamosvári 2020 [30]	*B. thailandensis*	C_7_, C_9_	C_7_, C_9_	-	C_7_, C_9_	-	-	-	-	C_5_, C_7_, C_9_, C_11_	C_7_, C_9_–C_11_	C_7_, C_9_	C_7_, C_9_–C_11_
Vial 2008 [31]	*B. ambifaria*	C_7_–C_9_	C_7_, C_9_, C_11_	-	-	-	-	-	-	C_5_–C_10_	C_5_–C_10_	-	-
Vial 2008 [31]	*B. thailandensis*	-	C_9_, C_11_	-	-	-	-	-	-	C_7_–C_9_	C_7_, C_9_, C_11_	-	C_8_–C_11_
Vial 2008 [31]	*B. pseudomallei*	-	-	-	-	-	-	-	-	C_7_	C_5_, C_7_–C_8_, C_10_–C_11_	-	C_6_–C_11_
Butt 2016 [43]	*B. pseudomallei*	C_7_, C_9_	-	-	-	-	-	-	-	C_7_–C_10_	C_7_–C_11_	-	C_8_–C_10_
Okada 2016 [44]	*B. thailandensis*	C_7_, C_9_	-	-	-	-	-	-	-	C_7_, C_9_	C_7_, C_9_, C_11_		C_7_–C_9_
Kim 2016 [22]	*Pseudoaltero-monas* sp.	C_3_–C_11_	-	-	-	-	-	-	-	-	-	-	-

HAQs—Hydroxy-alkylquinolones, AQNOs—Alkylquinolones-*N*-oxides, PQS—Pseudomonas Quinolone Signal, HMAQs—Hydroxy-methyl-alkylquinolones, HMAQNOs—Hydroxy-methyl-alkylquinolone-*N*-oxides.
